# Endocytosis and early endosome motility in filamentous fungi

**DOI:** 10.1016/j.mib.2014.04.001

**Published:** 2014-08

**Authors:** Gero Steinberg

**Affiliations:** Biosciences, University of Exeter, Stocker Road, Exeter EX4 4QD, UK

## Abstract

Hyphal growth of filamentous fungi requires microtubule-based long-distance motility of early endosomes. Since the discovery of this process in *Ustilago maydis*, our understanding of its molecular basis and biological function has greatly advanced. Studies in *U. maydis* and *Aspergillus nidulans* reveal a complex interplay of the motor proteins kinesin-3 and dynein, which co-operate to support bi-directional motion of early endosomes. Genetic screening has shed light on the molecular mechanisms underpinning motor regulation, revealing Hook protein as general motor adapters on early endosomes. Recently, fascinating insight into unexpected roles for endosome motility has emerged. This includes septin filament formation and cellular distribution of the machinery for protein translation.

## Highlights

• Early endosomes in filamentous fungi move along microtubules.
• In several fungi, endosome motility is mediated by kinesin-3 and dynein.
• In *Ustilago* and *Aspergillus*, a Hook protein was identified as a motor adapter on endosomes.
• Endosome motility serves sorting to the vacuole and distribution of mRNA and polysomes.
• Protein translation on endosomes appears to be required for septin filament assembly

**Current Opinion in Microbiology** 2014, **20**:10–18

This review comes from a themed issue on **Host–microbe interactions: fungi**

Edited by **Jay C Dunlap** and **Jean Paul Latgé**

For a complete overview see the Issue and the Editorial

Available online 15th May 2014

http://dx.doi.org/10.1016/j.mib.2014.04.001

1369-5274/© 2014 The Authors. Published by Elsevier Ltd. This is an open access article under the CC BY license (http://creativecommons.org/licenses/by/3.0/)

## Introduction

**Figure 1:**
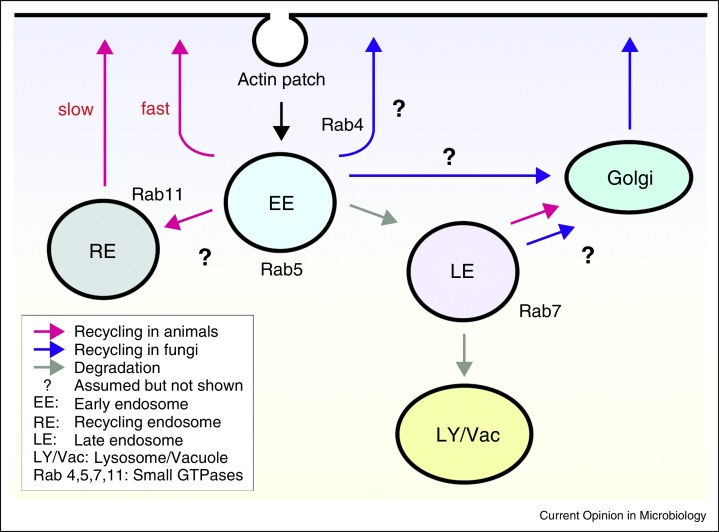
Schematic overview of endocytic pathways in animal cells and fungi. Endocytosis begins with the uptake of material into endocytic vesicles. In fungi, these vesicles are surrounded by F-actin [61, 73]. The first endocytic compartment is early endosomes (EEs), which carry the small GTPase Rab5 [5, 24]. In animal cells, recycling back to the plasma membrane involves EEs and the associated GTPase Rab4 (fast recycling) and recycling endosomes (RE) that carry the small GTPase Rab11 [74]. The pathways of recycling in fungi are not clear (indicated by ‘?’), but may involve late Golgi-associated membranes [75]. While travelling towards the vacuole/lysosome, EEs mature into late endosomes (LE), which involves a replacement of Rab5 by Rab7 [64•, 76].

The endocytic system comprises several compartments that receive cargo from the plasma membrane for processing and recycling back to the cell surface or its degradation in the lysosomes [1]. Early endosomes (EEs) are a central compartment in the endocytic pathway (Figure 1). They bind the small GTPase Rab5, which, together with its effectors, controls biogenesis, membrane fusion and microtubule-dependent motility in animal cells [2, 3, 4, 5]. Motility of animal endosomes supports endocytic sorting, but also participates in long-distance signal transduction, cytokinesis and cell migration [6, 7]. In the budding yeast *Saccharomyces cerevisiae* and the fission yeast *Schizosaccharomyces pombe*, long-distance transport along microtubules does not exist; this may relate to their small size. However, fungal endocytosis was described first in yeasts [8, 9, 10], and endocytic recycling supports their polar growth and survival [11, 12, 13]. In filamentous fungi, endocytosis supports hyphal growth (see Box 1; overview in [14, 15]), but in contrast to the yeasts, filamentous fungi contain EEs that move along microtubules. EEs were first described in *U. maydis* [16] (Table 1). A screen for morphological *U. maydis* mutants revealed a mutation in a soluble *N-ethylmaleimide sensitive* factor attachment protein receptor (SNARE), named Yup1. Temperature-sensitive Yup1^ts^ mutants showed defects in vacuolar sorting of FM4-64, a marker dye for tracking endocytic uptake in fungi [17, 18, 19, 20, 21], suggesting that the SNARE functions in the endocytic pathway. Indeed, Yup1 was located on organelles that stained rapidly with shortly after application of FM4-64 [16]. The organelles bind Phox-domains, which specifically interact with EE-characteristic lipids [22] and carry the EE-specific small GTPases Rab4 and Rab5 [23, 24, 25]. Thus, there is little doubt that the Yup1-positive organelles are EEs. Rapidly-moving Rab5-positive EEs were also described in *A. nidulans* [26] and *Neurospora crassa* [27]. Such organelles are thus a hallmark of filamentous fungi.

Box 1 **A historical perspective on endocytosis in filamentous fungi**

Filamentous fungal growth is characterized by apical extension of the hyphal cell. This process requires the constant supply of membranes and proteins, such as cell wall-forming enzymes, to the growing hyphal tip. The Spitzenkörper, an apical vesicle accumulation found in many fungal species [78, 79, 80, 81], is thought to be of key importance to tip growth. It was suggested that the Spitzenkörper consists of Goli-derived exocytic vesicles that fuse with the expanding apex to fuel tip growth [82]. This view was firstly challenged by the work of Hoffmann and Mendgen [18], who stained the Spitzenkörper of *Uromyces fabae* with the lipophilic marker FM4-64. At this time, the dye was already well-established as a tracer for the endocytic pathway in the yeasts *Saccharomyces cerevisiae* [21]. Therefore, staining of the Spitzenkörper with FM4-64 suggested that endocytic uptake of membranes participates in tip growth. Subsequent studies in *Ustilago maydis* [16, 20], *Magnaporthe grisea* [83], *Aspergillus nidulans* [19], *Neurospora crassa* and 6 additional other fungi [17] confirmed the uptake of FM4-64 into fungal cells. This strongly suggested that endocytsis is common in filamentous fungi. However, FM4-64 or other endocytic marker dyes were not taken up in some fungal species [84, 85]. In addition, fungal hyphae are continuously growing and endocytic vesicle uptake against the internal turgor pressure was difficult to envisage [85]. Thus, just a decade ago, the existence of endocytosis in fungal hyphae was a matter of debate [86]. To-date, elegant experimental studies in the fission yeast have shown that initial endocytic steps are slowed down by turgor pressure, but this is overcome by the energy-dependent assembly of the actin cytoskeleton [87]. In addition, overwhelming evidence coming from bioinformatic analysis of fungal genomes [86, 88], live observation of fluorescent proteins involved in endocytosis [35, 62], combined with mutant studies in *U. maydis*, *A. nidulans*, *A. oryzae, Candida albicans*, *Ashbya gossypii* and *N. crassa* [16, 58, 59, 60, 61, 62, 63, 89, 90, 91, 92, 93, 94•, 95, 96], leaves no doubt that endocytosis and endocytic recycling is of central importance for hyphal growth (for more comprehensive overview see [14, 15]).

**Table 1:** Scientific milestones in endocytosis research in filamentous fungi

Contribution	Fungal system	Reference
First report of endocytosis in filamentous fungi	*Uromyces fabae*	[18]
First report on a role of microtubules in fungal endocytosis	*Ustilago maydis*	[20]
Identification of motile early endosomes that move along microtubules; first indication of a role of endocytic recycling in fungal morphology	*Ustilago maydis*	[16]
Establishment of FM4-64 as a general tracer for endocytosis in filamentous fungi	*Neurospora crassa* and 9 other species	[17]
First description of fungal kinesin-3 and its role in opposing dynein in motility of early endosomes	*Ustilago maydis*	[37]
Identification of the apical MT plus end as a “dynein loading zone” for binding EEs to the retrograde motor	*Ustilago maydis*	[36]
First report of a role of endocytosis in receptor recycling during fungal pathogenicity	*Ustilago maydis*	[24]
Identification of an apical collar-like region of endocytic uptake and recycling	*Aspergillus nidulans*	[60, 61, 62]
Discovery that Ascomycete kinesin-3 utilizes a subset of detyrosinated (less dynamic) microtubules	*Aspergillus nidulans*	[40]
Report on the down-regulation of a plasma membrane transporter by substrate-induced endocytosis	*Aspergillus nidulans*	[95]}
First insight into EE-to-motor attachment by reporting a role of dynactin subunit p25	*Aspergillus nidulans*	[51]
First report of a role of EEs in transporting mRNA	*Ustilago maydis*	[66^•^]
First report on a role of retrograde motility in early-to-late endosome maturation	*Aspergillus nidulans*	[64^•^]
First description of an up-regulation of actin-patch dynamics and associated endocytosis in hyphal versus yeast-like growth	*Candida albicans*	[97^•^]
Discovery of clathrin-independent endocytosis	*Candida albicans*	[94^•^]
Discovery of a biological role of EE-associated translation	*Ustilago maydis*	[69^••^]
Identification of a role of bidirectional EE motility in distribution the machinery for protein translation	*Ustilago maydis*	[56^••^]
Identification of Hook proteins as adapters for EE motors	*A. nidulans*/*U. maydis*	[53^••^]/[52^••^]

*Note:* Reports on eisosomes, which were implied in fungal endocytosis [98], are not included as their suggested role as endocytic portals is a matter of debate [99].

## The molecular machinery for early endosome motility

### Endosome motility is a microtubule-dependent process

Microtubules are polymers of tubulin dimers that support long-range motility of organelles and vesicles in eukaryotic cells. Numerous studies have suggested that Golgi-derived secretory vesicles support hyphal growth [28], and they are likely to be transported to the hyphal tip by motors [29]. This notion was supported by the discovery of the motor protein kinesin-1 in Asco-, Basidio- and Zygomycetes [30, 31, 32, 33]. Subsequent studies confirmed a role for this microtubule-dependent motor in delivering chitin synthase-containing secretory vesicles [34], but also indicated a role in endocytic uptake of the marker dye Lucifer Yellow [20]. This suggested that microtubules support endocytosis. Indeed, the Yup1-positive EEs were shown to move bi-directionally along microtubules [16]. A similar behaviour was reported in *A. nidulans* [26], *A. oryzae* [35] and *N. crassa* [27].

### EE loading onto dynein occurs at microtubule plus-ends

Microtubules have an intrinsic polarity, with most polymerization occurring at the plus-end, whereas the minus-end is usually blocked by a nucleation site. Molecular motors utilise this polarity and kinesins move their cargo to plus-ends, whereas dynein transports organelles towards minus-ends. In filamentous fungi, EE motility is bi-directional and is mediated by kinesin-3 and dynein [27, 36, 37, 38, 39•, 40]. Dynein accumulates at the plus-ends of microtubules [36, 41] and the motor kinesin-1 is required for this plus-end targeting [36, 42]. EE binding to dynein, for retrograde transport to sub-apical parts of the hyphal cell, occurs mainly at the microtubule plus-ends [36, 38, 43, 44]. This led to the idea that the microtubule plus-end is a ‘dynein loading zone’ [36]. However, it is important to note that dynein loading also takes place in sub-apical parts of the cell [44]. It was suggested that dynein accumulation at plus-ends increases the efficiency of dynein interacting with EEs [38]. Indeed, quantitative light microscopy studies demonstrated that 50-60 dynein motors concentrate at apical plus-ends. Experimentally lowering this number, by interfering with the mechanism of anchorage, reduced the loading of EEs to the motor [43]. Under these conditions, the number of EEs that run too far and ‘fall off’ at the microtubule end increases ∼5 times. This suggests that the accumulation of dynein at plus-ends serves as a ‘buffer stop’ that keeps the organelles on the microtubule track [43]. This concept assumes a stochastic interaction of dynein and EEs. However, additional dynein regulators, such as NudF/Lis1 [45], have been shown to be required for retrograde EE motility [36, 39•]. Fungal Lis1 concentrates at plus-ends [36, 41], but does not co-travel with retrograde moving dynein or EEs [36, 39•]. This led to the suggestion that Lis1 is an initiation factor for retrograde EE motion [39^•^].

### Kinesin-3 is the motor for tip-directed motility of EEs

The human genome encodes 45 kinesin motors of which 8 belong to the kinesin-3 family [46]. Kinesin-3-like motors are absent from the genomes of *S. cerevisiase* and *Schizosaccharomyces pombe*. This coincides with the absence of microtubule-based EE motility in these yeasts. Early studies in *U. maydis* identified kinesin-3 as the anterograde motor for EE motility [36, 37]. Subsequently, kinesin-3 and its role in EE motility was discovered in *A. nidulans* [39•, 40] and *N. crassa* [27]. While kinesin-3 appears to be the major motor for EE motility, a role in secretion was also reported [47]. Kinesin-3 opposes dynein in mediating bi-directional motility of EEs. In animal cells [48] and *A. nidulans* [39^•^], dynein and kinesin motors are bound simultaneously to the cargo. In *U. maydis*, however, anterograde kinesin-3-delivered EEs do not bind dynein [44]. In this fungus, a turn in EE-transport direction is achieved in two ways. Firstly, each EE carries ∼3–5 kinesin-3 motors; this may allow ‘hopping’ from one microtubule to another, without interrupting transport. As most EE motility occurs along microtubule bundles, consisting of anti-polar oriented microtubules [49], ‘hopping’ causes a change in transport direction. Secondly, kinesin-1 delivers dynein to the microtubule plus-end [36], where it accumulates and from where it is released to travel 'freely' towards minus-ends [43]. This dynein is competent to bind anterogradely-moving EEs, which turns the transport direction from anterograde to retrograde [44]. This is achieved by single dynein motors that probably inactivate the excess of kinesin-3 (see below). Finally, it should be noted that Ascomycete kinesin-3 moves cargo along a subset of detyrosinated, less dynamic microtubules [27, 40]. The biological reason for this merits further investigation.

### Hook proteins link motors to EEs

Motor proteins often interact with their cargo membrane via adapter complexes, which control the affinity of the motor for the cargo and regulate the direction of transport [50]. In animal cells, several adapter complexes for dynein and kinesin-1 have been described, but no adapter for kinesin-3 is known. In fungi, our knowledge is restricted to a single report, which shows that the p25 subunit of the dynactin complex links dynein to EEs [51]. However, neither a kinesin-3 binding partner on EEs, nor any regulatory protein has been described. To identify such regulatory machinery for bi-directional EE motility, genetic screening was undertaken in *A. nidulans* and *U. maydis*, leading to the simultaneous description of Hook proteins as an adapter for dynein on EEs [52••, 53••]. Hook proteins were first identified in fruit flies and are intensively studied in human cells, where they appear to link organelles to microtubules [54, 55]. In fungi, *hook* null mutants are defective in retrograde EE motility, as the organelles are no longer able to bind dynein [52••, 53••]. It was shown biochemically [53^••^] and by live cell imaging [52^••^] that the N-terminal part of fungal hook proteins (HookA in *A. nidulans*, [53^••^]) and Hok1 in *U. maydis*, [52^••^]) interact with the dynein/dynactin complex, whereas the C-terminal region binds to EEs. In *A. nidulans*, EE motility requires the Rab5-GTPase RabB [57]. Whether hook proteins are anchored to EE membranes via Rab5 GTPases remains to be seen.

**Figure 2:**
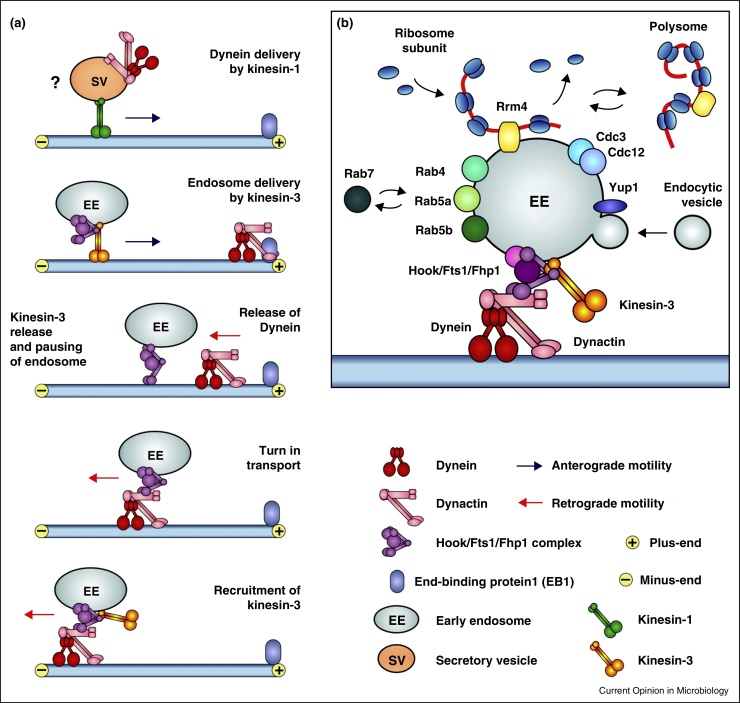
**(a)** Schematic illustration of the role of the Hook-complex in coordinating motor attachment in *U. maydis*. EE motility depends on three motors, kinesin-1, kinesin-3 and dynein. Kinesin-1 delivers the dynein/dynactin complex to microtubule plus-ends near the hyphal apex. This may involve secretory vesicles, though experimental evidence for this is missing (indicated by ‘?’). Kinesin-3 binds to EEs via the Hook-complex, consisting of Hok1, and homologues of the human oncogene FTS and FHIP, both shown to interact with Hook in humans [77]. Dynein accumulates at plus-ends via a stochastic traffic jam and a specific interaction of the p150^glued^ subunit of dynactin and EB1 [43]. The motor is released from the microtubule plus-ends and can bind to EEs during its journey towards the minus-end. EEs usually pause prior to rebinding, which coincides with a kinesin-3 release [52^••^]. Binding of dynein initiates retrograde motility, which persists while kinesin-3 is recruited back onto the EEs. **(b)** The current knowledge of proteins binding to EEs in *U. maydis*. The Hook-complex, consisting of Hok1, Fts1 and Fhp1 serves as an adapter for kinesin-3 and dynein [52^••^]. Yup1 is a putative endosomal SNARE [16] that mediates fusion of transport vesicles the organelles. This function is crucial for endocytic recycling, and *yup1*^ts^ mutants are defective in morphology [16] and receptor recycling [24]. The small GTPases Rab5a, Rab5b and Rab4 locate to EEs [24, 56••], but their cellular role is not known. Rrm4 is an EE-associate RNA-binding protein [65, 66•] that anchors mRNA and associates ribosomes to EEs [56^••^]. The entire polysome can ‘fall off’ or rebind to moving EEs, which evenly-distributes the translation machinery [56^••^]. In addition, EEs have recently implied in assembly of septin filaments (Cdc3 and Cdc12) [69^••^].

Surprisingly, Hok1 in *U. maydis* also controls kinesin-3 binding to EEs. A short and highly conserved part of the N-terminal coiled-coil region of Hok1 is crucial for this, but also for dynein binding to EEs. This suggests that an unidentified Hok1-binding protein bridges between Hok1 and both motors. In *U. maydis*, homologues of the human proteins FTS and FHIP bind to Hok1 and localize to EEs [52^••^]. The precise role of both proteins in both humans and fungi remains to be unmasked, but they may support motor attachment of Hok1 on EEs. The ability of Hok1 to bind both motors suggests that the Hook/FTS/FHIP complex serves as a coordinator for EE bi-directional motility. Hok1 may control attachment of both kinesin-3 and dynein to the cargo, thereby regulating the transport direction (Figure 2a, [52^••^]). Interestingly, hook proteins, like kinesin-3, are not present in *S. cerevisiae* and *S. pombe*. As both are found in filamentous fungi and in animals [52^••^], they appear to form a functional pair, required for long-range, bi-directional motility of cargo along microtubules.

## The cellular role of endosome movement

### EE motility and the endocytic pathway

Endocytic recycling near the hyphal apex supports fungal tip growth in *A. nidulans*, *A. oryzae*, *N. crassa and Ashbya gossypii* [15, 58, 59, 60, 61, 62, 63] and receptor exposure during early pathogenic development of *U. maydis* [24]. Recycling appears to depend on fusion of transport vesicles with the EE [16, 24], which are also thought to be involved in sorting cargo to the subapical vacuole for degradation. Thus, it is likely that EE motility shuttles the organelles between the expanding tip and the subapical vacuolar system. However, in *A. oryzae* uptake of uric acid-xanthine permease, from the plasma membrane and delivery to vacuoles, occurs in the absence of EE motility [35]. While this finding questions a role of EE motility in vauolar sorting, a recent report by Penalva [64^•^] demonstrates that retrograde motility is required to allow fusion of late endosomes with vacuoles. In *A. nidulans*, EEs convert into late endosomes and, finally, subapical vacuoles [57]. During this maturation, Rab5 (RabB/A in *A. nidulans*) is replaced by Rab7 (RabS in *A. nidulans*). In temperature-sensitive dynein mutants, retrograde EE motility is blocked and Rab7-positive late endosomes/vacuoles, stained with the blue dye CMAC (7-Amino-4-Chloromethylcoumarin), cluster near the hyphal tip. These results suggest that endosomes mature and fuse to form vacuoles as they move away from the tip [64^•^]. A role for EE motility in sorting to the vacuole was also reported in *U. maydis* [56^••^]. When binding of dynein was impaired in *hook* deletion mutants (see above), or when the dynein heavy chain was inactivated in temperature-sensitive mutants, EEs formed clusters at the hyphal tip and delivery of FM4-64 to the vacuoles was blocked [56^••^]. Surprisingly, FM4-64 concentrated in a ‘cloud’ of Rab7-positive small vesicles that were not stained with CMAC [56^••^], while vacuole organization remained largely unaffected. This raises the possibility that EE motility is required to allow efficient fusion of Rab7-positive transport vesicles with the vacuole.

### EEs deliver mRNA to the cell poles

An unexpected insight into a cellular role for EE motility in fungi was provided by work on RNA-binding proteins. In *U. maydis*, the putative RNA-binding protein Rrm4 moves along microtubules [65]. This motility requires dynein and kinesin-3 [66^•^], motors that were shown previously to mediate bi-directional EE motility [36, 37]. Indeed, co-localization studies and the use of temperature-sensitive Yup1 mutants revealed that Rrm4 ‘hitchhikes’ on moving EEs [66^•^]. Rrm4 binds various mRNAs, including those encoding the ubiquitin fusion protein Ubi1 and the small G protein Rho3 [67]. It was therefore suggested that EE motility towards the cell ends supports polar delivery of Rrm4-bound RNA [68]. However, *in vivo* observation of fluorescent Rrm4 particles demonstrated that Rrm4 motility is bi-directional and that extended runs of these particles are rare [67]. These results raised doubt about an exclusive role of EE motility in long-distance transport of mRNA. A recent paper by Feldbrügge [69^••^] provides an unexpected answer to this conundrum. It shows that the mRNA of the septin *cdc3* and *cdc12* is actively-translated on moving EEs and both proteins bind to EEs. Interestingly, the presence of Cdc3 on EEs requires Cdc12, suggesting that assembly of septin complexes occurs on EEs. This is important for proper septin filament formation and, in turn, hyphal growth.

### EEs distribute the machinery for protein translation

A second study in *U. maydis* confirms translation of mRNA on moving EEs, but it came to the conclusion that ribosome binding to mRNA serves to distribute polysomes throughout the cell [56^••^]. Ribosomal subunits are produced constantly in the nucleus and are thought to distribute by diffusion. However, the visco-elastic properties of the cytoplasm limit passive diffusion of large particles and vesicles [70]. Indeed, the use of photo-activatable ribosomal proteins in *U. maydis* revealed that passive diffusion is too slow to ensure even cellular distribution of the ribosomal subunits [56^••^]. Mathematical modelling indicated that an active transport component is required to spread ribosomes through the fungal cell. Indeed, ribosome distribution was impaired in kinesin-3 and dynein mutants, but was also involved the RNA-binding protein Rrm4. This suggests that ribosome distribution involves EE motility. Surprisingly, quantitative live-cell imaging of ribosomes demonstrated that entire polysomes assemble on moving EEs [56^••^]. While travelling, the ribosomes are translationally active, thereby binding Rrm4-anchored mRNA to be spread within the cell. This is achieved by random release, but also rebinding, of entire polysomes from the EEs. Thus bi-directional EE motility constantly “stirs” the translation machinery in the cell, thereby adding an active transport component to the passive diffusion of ribosomes in the cytoplasm. Null mutants in *rrm4* and the kinesin-3 gene *kin3* exhibit a very similar phenotype [56^••^], suggesting that distributing of the translation machinery is a major role of EE motility in *U. maydis.*

## Conclusion

The existence of endocytosis and EEs in fungi was a matter of debate until relatively recently. Today, it is well-established that endocytic recycling supports fungal morphology and that EEs rapidly move within the fungal cell. Intensive research in *U. maydis* and *A. nidulans* has elucidated the molecular machinery underlying EE motility and has provided insight into fundamental principles of motor co-operation in membrane trafficking [71]. Most recently, genetic screening led to the identification of novel kinesin-3 adapters and this discovery will most likely stimulate future research in mammalian systems. We are also beginning to understand why EEs move. While a role for retrograde EE motility in sorting to the vacuole is expected, its function in distributing mRNA, supporting protein translation ‘on the move’ and spreading the translation machinery is surprising, yet potentially of great significance for growth and function of fungal hyphae. New biological roles for EEs in fungi will inevitably be discovered. Indeed, indirect evidence suggests aflatoxin biosynthesis in *Aspergillus parasiticus* involves EEs [72] (J.E. Linz, pers. communication). Thus, fungal EEs may provide a ‘platform’ to integrate several cellular pathways and their respective functions (Figure 2b), a concept first suggested in animal systems [7]. Highly complementary work on EE function and motility in the model systems *U. maydis* and *A. nidulans* has turned this pioneering field into a fast-moving research area in fungal cell biology (Table 1). Future discoveries in this area promise exciting and unexpected insight into how fungal cells function. Furthermore, work in fungal systems will almost certainly help unveil fundamental principles of membrane trafficking in eukaryotic cells.

## References and recommended reading

Papers of particular interest, published within the period of review, have been highlighted as:

• of special interest
•• of outstanding interest
